# Supplementary material for: TGFβ-induced degradation of TRAF3 in mesenchymal progenitor cells causes age-related osteoporosis
Source: Nat Commun. 2019 Jun 26;10:2795. doi: 10.1038/s41467-019-10677-0 (PMC6595054; doi:10.1038/s41467-019-10677-0)
Supplement: Supplementary file 1 — Supplementary Information [file 41467_2019_10677_MOESM1_ESM.pdf]

Supplementary Figure 1

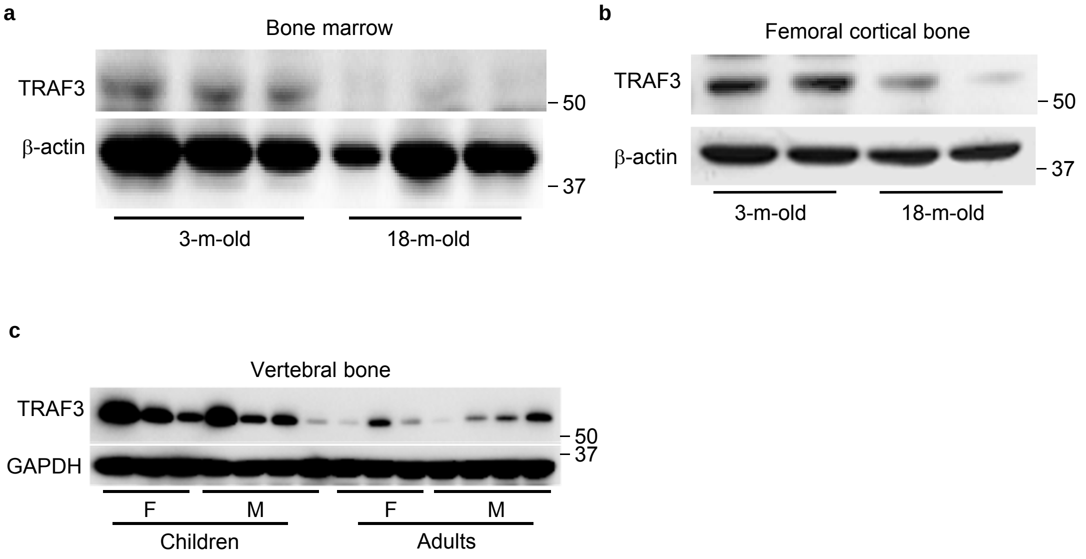

Supplementary Figure 1. TRAF3 protein levels decrease in both bone and BM during aging. Total protein was extracted separately from tibiae (including BM), BM flushed from femora, and femora (cortical bone without BM) from young (3-m) and old (18-m) WT mice (a and b) and from vertebral spinous processes (containing bone and BM) from 3 female (F) and 4 male (M) children and adults (c). Levels of TRAF3/GAPDH in the protein lysates were tested by WB. The data are representative of multiple samples from young and old mice and humans.

Supplementary Figure 2.

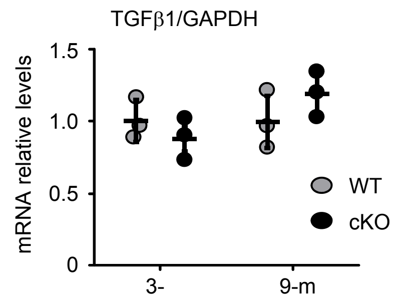

Supplementary Figure 2. mRNA levels of TGFβ1 in BdMPCs from 3- and 9-m-old cKO mice and WT littermates. n=3/group.

Supplementary Figure 3.

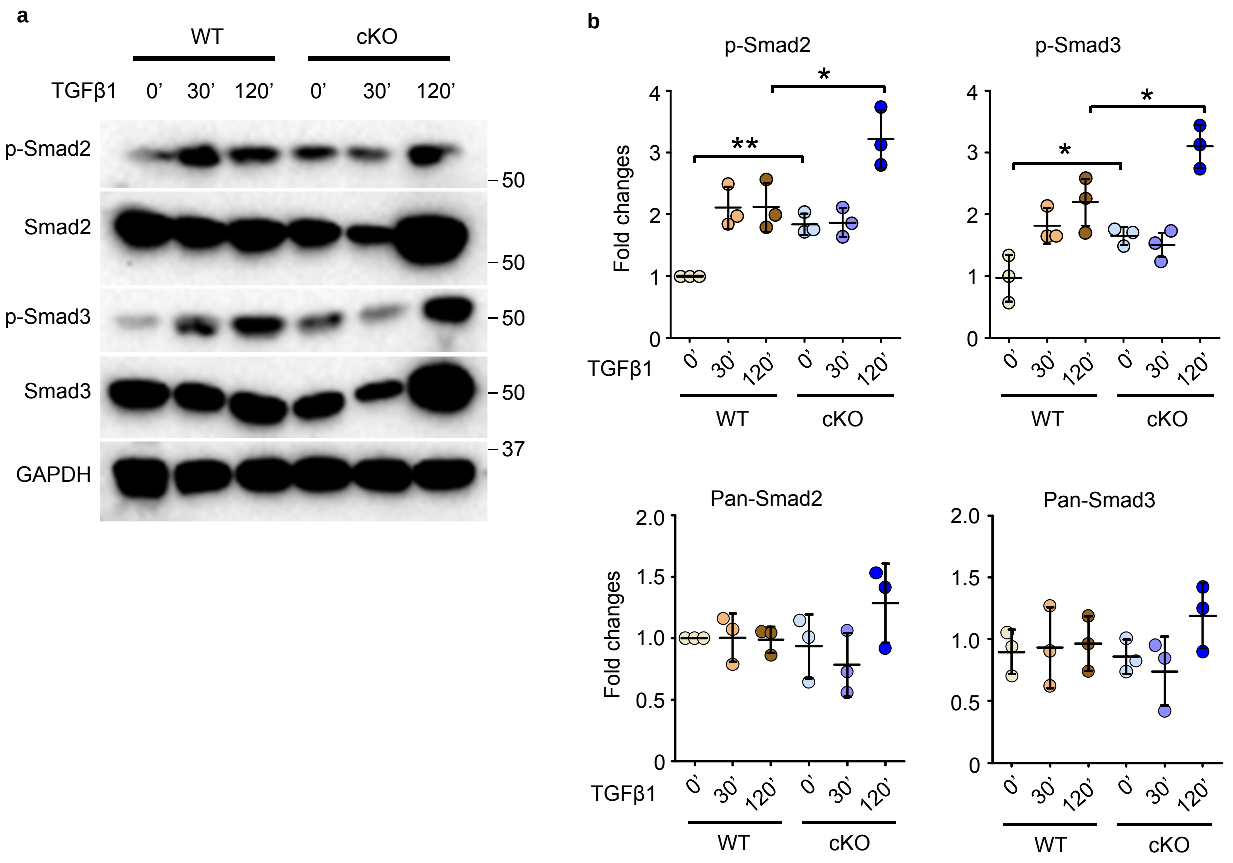

Supplementary Figure 3. BdMPCs (10^6 cells) from 3-m-old WT and cKO mice were seeded in α-MEM with 10% FBS for 1 day in 100 mm dishes and treated with TGFβ1 (2ng/ml) for 0 (vehicle), 30 and 120 min before harvesting for protein analysis by WB. Densitometric analysis of p-Smad2 and p-Smad3 was compared to GAPDH and standardized by the average level of p-Smad2 or p-Smad3 in WT Ctrl cells (0 min). Each dot represents a value generated from an individual experiment in dot-plots. *p<0.05; **p<0.01.

Supplementary Figure 4.

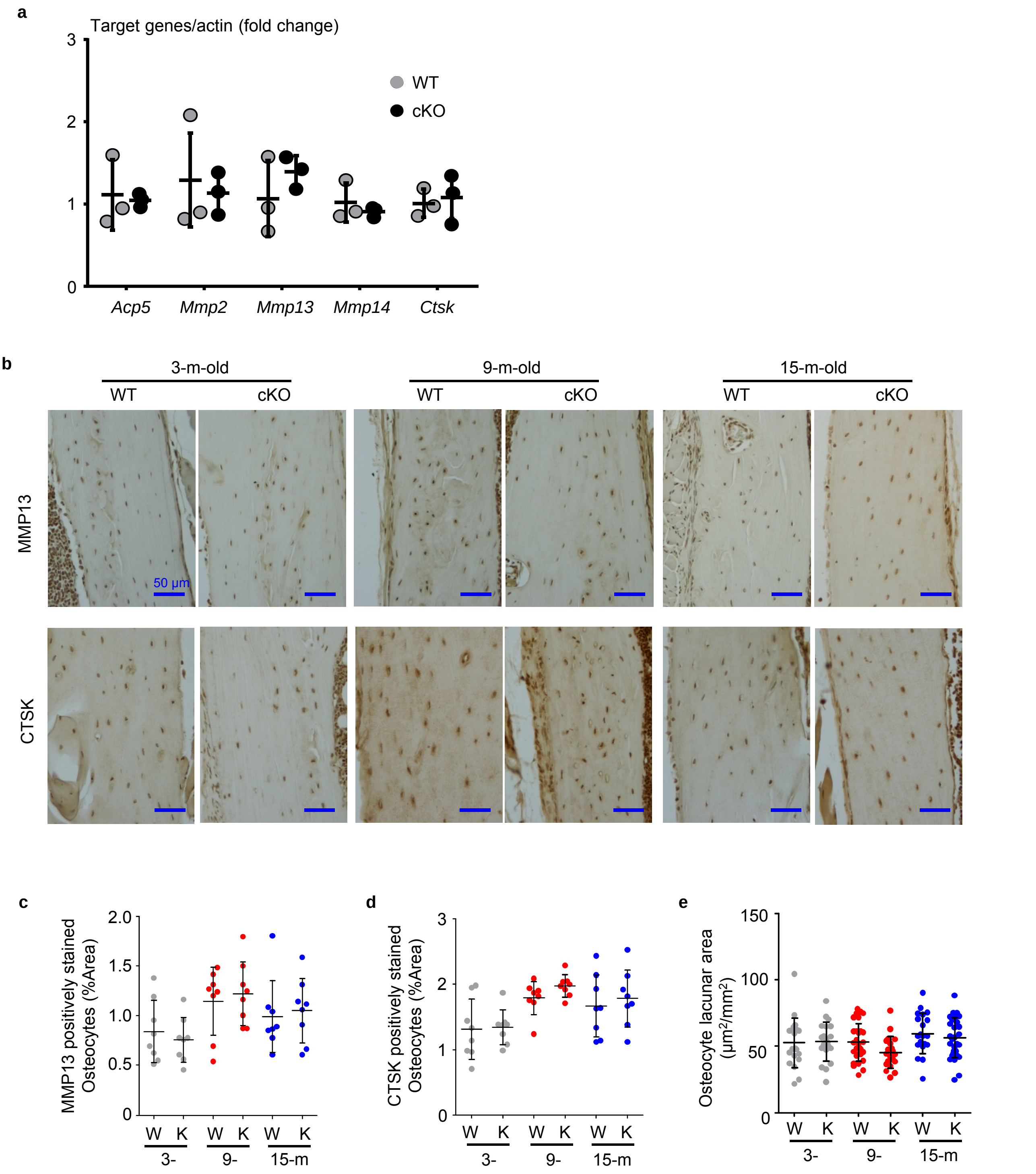

Supplementary Figure 4. Remodeling of perilacunar/canalicular matrix is not impaired in TRAF3 cKO mice. (**a**) Total RNA was extracted from tibiae of 15-m-old WT and TRAF3 cKO mice. mRNA expression levels of TRAP (*Acp5*), matrix metalloproteinase 2 (*MMP2*), *MMP13*, *MMP14* and cathepsin K (*CTSK*) were tested using real-time PCR. (**b**) Immunostaining for MMP13 and CTSK was performed on decalcified tibial sections from 3-, 9- and 15-m-old WT (W) and TRAF3 cKO (K) mice. The areas of (**c**) MMP13$^+$ and (**d**) CTSK$^+$ osteocytes were quantified and expressed as % total bone area. (**e**) Osteocyte lacunar area per mm^2 bone area was quantified on H&E sections (n=8 mice/group).

Full Blots

Fig. 1(c)

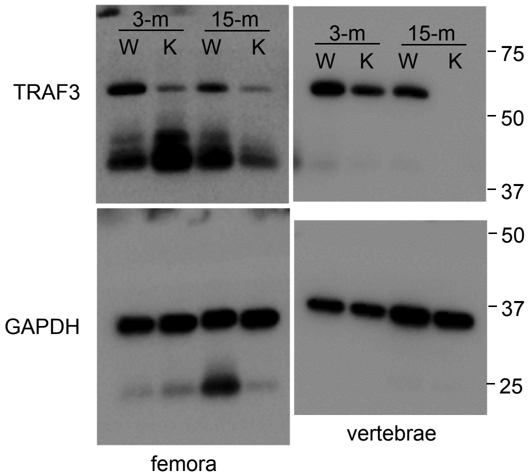

Fig. 3(b) &
Supplementary Fig. 2(c)

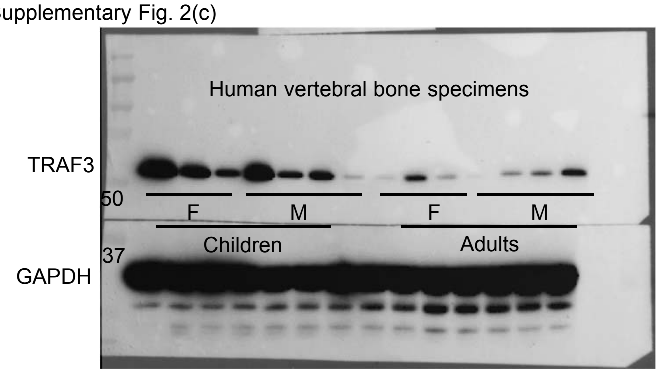

Fig. 3(d)

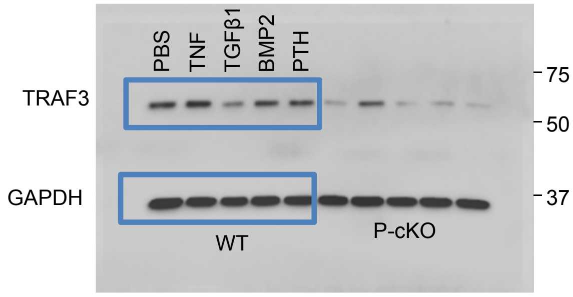

Fig. 3(h)

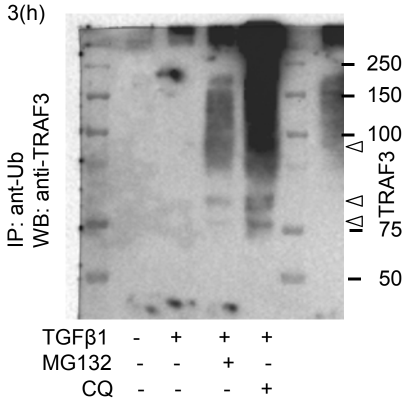

Fig. 3(i)

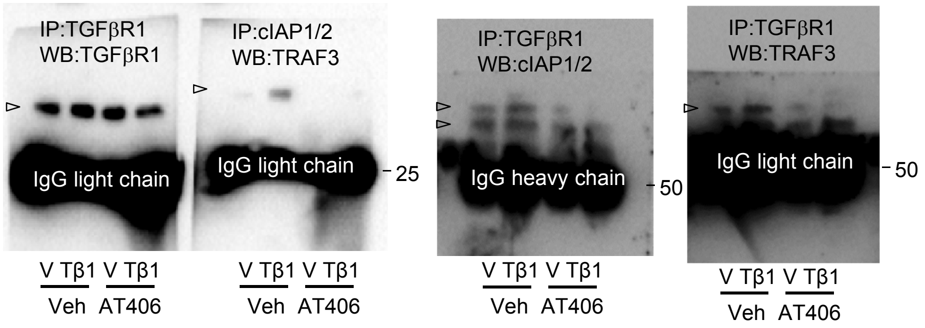

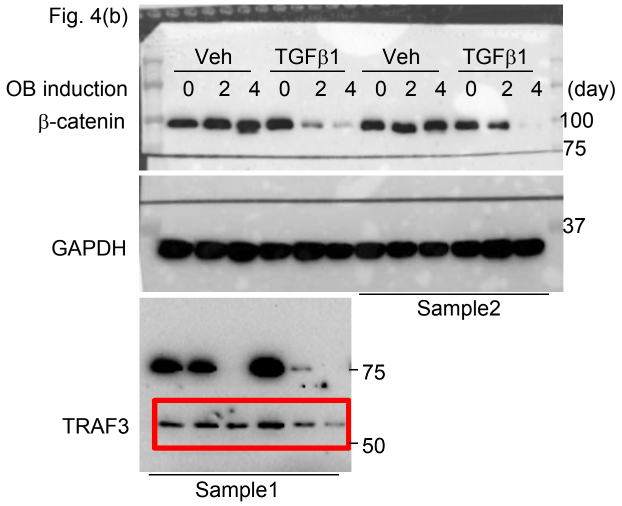

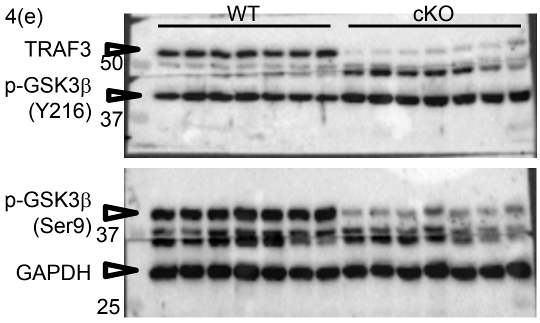

Full Blots

Fig. 4(f)

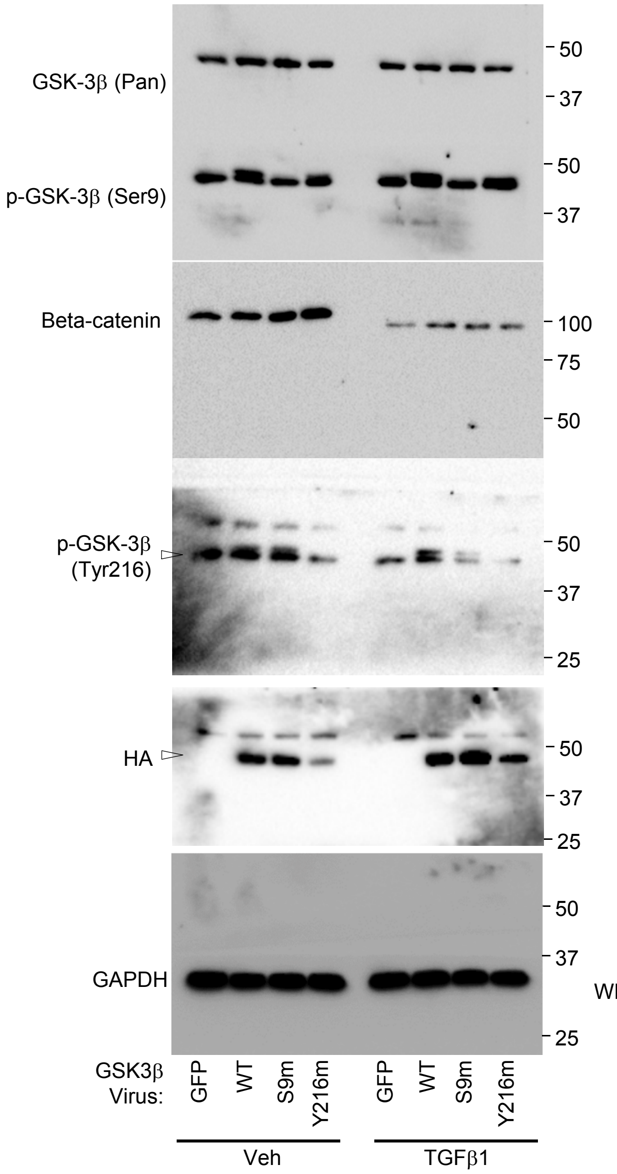

Fig. 4(g)

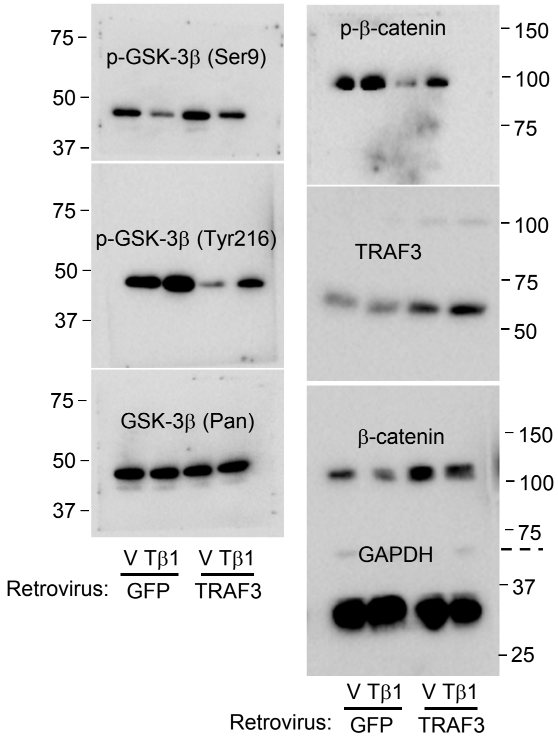

Fig. 4(h)

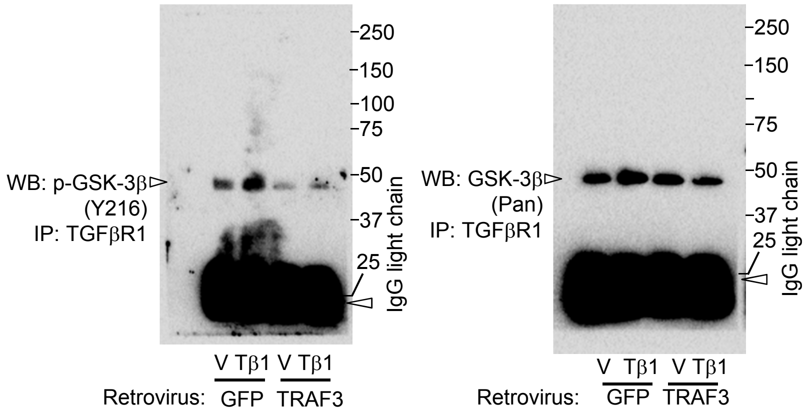

Fig. 4(k)

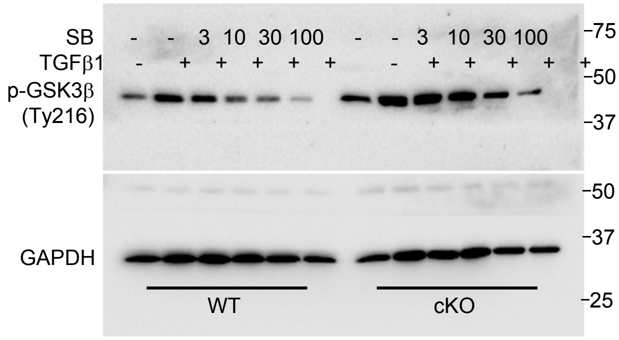

Fig. 5(c)

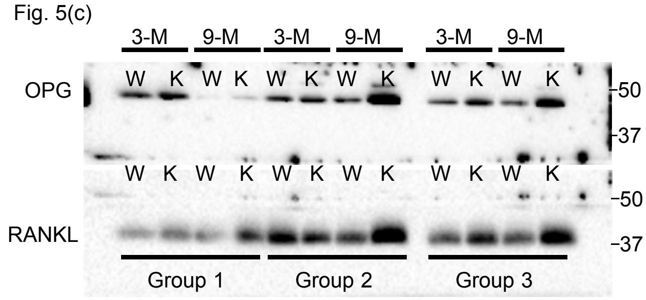

Full Blots

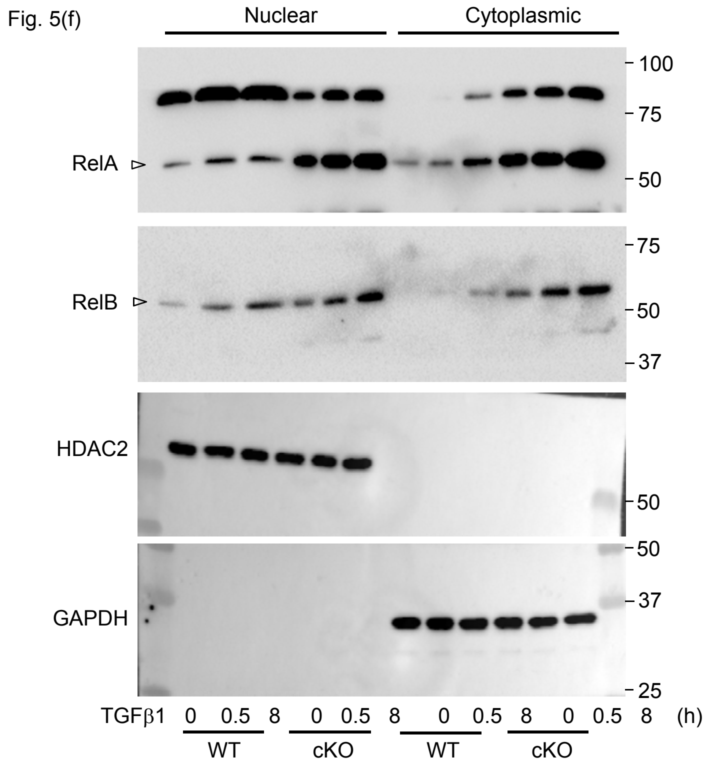

Fig. 5(f)
Nuclear
Cytoplasmic
RelA
RelB
HDAC2
GAPDH
100
75
50
75
50
37
50
50
37
25
TGFβ1 0 0.5 8 0 0.5 8 0 0.5 8 0 0.5 8 (h)
WT
cKO
WT
cKO

Flow Gating Strategies

Fig. 2(**e**)

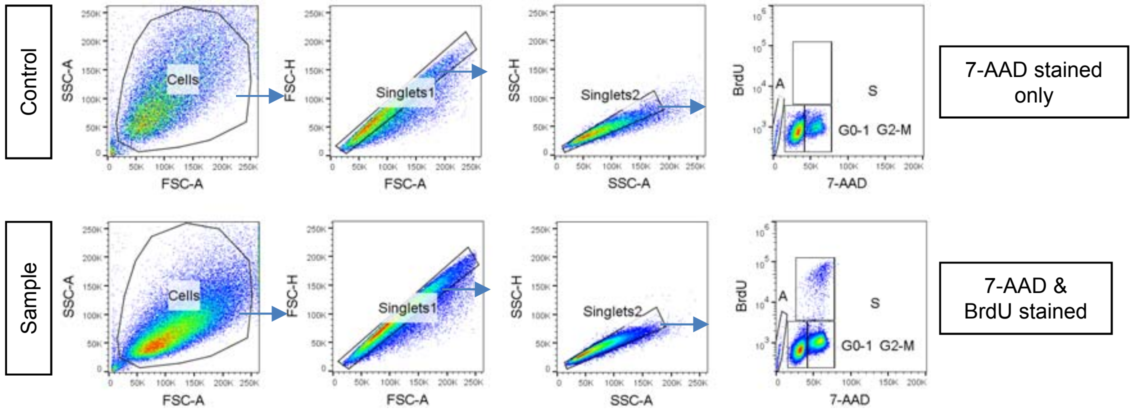

Gating strategies used for flow analysis in Fig. 2(e).

Fig. 6(**e**)

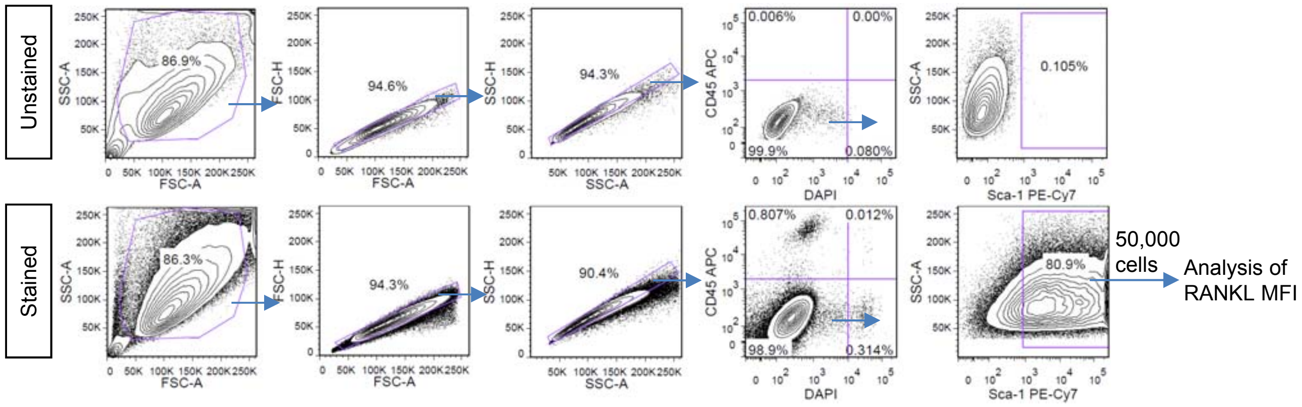

Gating strategies used for flow analysis in Fig. 6(e).
